# What It Might Achieve

**Published:** 1920-10-30

**Authors:** 


### A STATE NURSING SERVICE.

### What it Might Achieve.

Even assuming that a State Nursing Service is as far off as Mars, it can do no serious harm to set out in broad outline what would naturally ensue as a consequence if the apparently impossible became a *fait accompli.* The way of reform is long and weary, and people are slow to convince. I wonder how many hospital readers remember receiving letters from William Willett when he first conceived the idea of changing the clock in order to save the daylight. I received more than one, and I well remember how amused I was at his dreams. By his persistent preaching he has saved the world many millions. But it took years to convince the world that such saving could be effected by so simple a device.

Now suppose that all the mountains were crossed and that a State Nursing Service were established, how would existing institutions be affected? The man-in-the-street must satisfy himself on this point before expressing approval or disapproval.

### The Nursing Profession.

The nursing profession would find itself translated automatically to a higher plane, and the change would immediately be felt by all ranks, and also by that not less important class outside the ranks, candidate probationers. Instead of matrons having to go out into the highways in quest of good material there would be a long queue, and the majority of the waiters would present first-class credentials. The tradition that the nursing profession should be a suitable service for gentlewomen would revive, for, unfortunately, save in the premier institutions, this has been falling into desuetude. As in all professions candidates would require to pass an entrance examination showing that a reasonable educational standard had been observed, and the addition of such optional subjects as needlework and housecraft would guarantee that the after-school interval had not been waste. "To him that hath shall be given" would appear to be an inexorable, if not an altogether equitable, law applying to both worlds. There is no room for doubt that for such an uplifted nursing world there would be many valuable prizes. No Minister of Health could avoid offering the State nurse all such higher appointments within his purview as Health Visitor. There would be no fear of staleness in the hospital service. On the contrary, there would be a steady flow of promotion.

The main function of a hospital is to heal the sick. But a subsidiary function of almost, if not altogether, equal importance is that of training or making the physician and the surgeon. The well-to-do public forget this. They do not realise that the marvels of medical science of which they gratefully avail are hospital products pure and simple, and that these institutions are staggering. If it so happen that they should survive the storm without being thrown on the rates, an immense relief to those responsible for their finances would be a free nursing supply. The idea is not new. I recently came across an article in an American magazine giving an account of the founding, some forty or fifty years ago, of a New England hospital, and considerable emphasis was laid on the fact that the nurses were not a charge on the hospital funds, but were paid and maintained from another source. I can see no objection to such an arrangement if it were possible. Discipline could not suffer, as hospital and nurse would be interdependent, and continuity of employment would be necessary for salary and pension.

Moreover, the hospital could, without difficulty, charge probationers a fee for training, just as do all training establishments, and the Minister of Health might be persuaded to grant a subsidy *per caput* as he does now to recognised schools for training women as health visitors.

### The Opposition.

I note the editorial finger pointed to certain quarters from which opposition might be expected. I wonder if we could have a few of those Goliaths out, so that we might take their measure. Of course, I am aware that I have so far made no reference to the man with the purse. Somebody would have to pay. There is a great deal to be said on this subject, apart from the ultimate fact. For example, if the Treasury had to be the medium, tempestuous weather might be looked for. But even such a Goliath might be slain.

Remember that six millions of women have Parliamentary votes, and that there are very few women who do not appreciate the value of a trained nurse. And also remember that owing to the unfavourable conditions of employment at present prevailing, and likely to continue to prevail, the high-class nurse is threatened with extinction. Until somebody comes forward with a scheme more likely to succeed in saving the situation I venture to claim that that of a State Nursing Service holds the field.

IERNE.

